# Geographic disparities in colorectal cancer survival

**DOI:** 10.1186/1476-072X-8-48

**Published:** 2009-07-23

**Authors:** Kevin A Henry, Xiaoling Niu, Francis P Boscoe

**Affiliations:** 1 New Jersey Department of Health & Senior Services, New Jersey State Cancer Registry, Cancer Epidemiology Services, Trenton, New Jersey, USA; 2 New York State Cancer Registry, New York State Department of Health, Albany, NY, USA

## Abstract

**Background:**

Examining geographic variation in cancer patient survival can help identify important prognostic factors that are linked by geography and generate hypotheses about the underlying causes of survival disparities. In this study, we apply a recently developed spatial scan statistic method, designed for time-to-event data, to determine whether colorectal cancer (CRC) patient survival varies by place of residence after adjusting survival times for several prognostic factors.

**Methods:**

Using data from a population-based, statewide cancer registry, we examined a cohort of 25,040 men and women from New Jersey who were newly diagnosed with local or regional stage colorectal cancer from 1996 through 2003 and followed to the end of 2006. Survival times were adjusted for significant prognostic factors (sex, age, stage at diagnosis, race/ethnicity and census tract socioeconomic deprivation) and evaluated using a spatial scan statistic to identify places where CRC survival was significantly longer or shorter than the statewide experience.

**Results:**

Age, sex and stage adjusted survival times revealed several areas in the northern part of the state where CRC survival was significantly different than expected. The shortest and longest survival areas had an adjusted 5-year survival rate of 73.1% (95% CI 71.5, 74.9) and 88.3% (95% CI 85.4, 91.3) respectively, compared with the state average of 80.0% (95% CI 79.4, 80.5). Analysis of survival times adjusted for age, sex and stage as well as race/ethnicity and area socioeconomic deprivation attenuated the risk of death from CRC in several areas, but survival disparities persisted.

**Conclusion:**

The results suggest that in areas where additional adjustments for race/ethnicity and area socioeconomic deprivation changed the geographic survival patterns and reduced the risk of death from CRC, the adjustment factors may be contributing causes of the disparities. Further studies should focus on specific and modifiable individual and neighborhood factors in the high risk areas that may affect a person's chance of surviving cancer.

## Background

In the past several years, there has been significant progress in reducing colorectal cancer (CRC) incidence and mortality rates in most US population groups [1]. Despite this progress, an unequal cancer burden is borne by blacks, relative to whites, and by individuals of lower socioeconomic position. These groups have higher incidence and mortality rates, lower survival rates, and greater percentages diagnosed at advanced stage [2,3]. Differences in CRC survival have been consistently observed in these groups even after adjusting for stage at diagnosis, a significant prognostic factor [4,5]. Survival disparities have been attributed to differences in individual and area-based socioeconomic factors, differences in access to and receipt of quality treatment, and post treatment follow-up, [3,6-9], and/or from differences in comorbidity [4].

Geographic disparities in survival have also been observed in several international and US studies for several cancer sites including colorectal cancer [10-23]. Knowing whether cancer survival varies geographically is especially relevant since area-based physical, social and behavioral factors can assist or impede patient survival. It is also relevant because health care is often delivered locally, and, therefore, the identification of areas with significantly better or worse survival outcomes may, for example, reflect access to and quality of care. Some factors offered as potential explanations for geographic variation in survival include regional or local distributions of patient characteristics, (race/ethnicity) tumor characteristics (stage), modifiable lifestyle factors (smoking, diet, exercise) [24], area-based characteristics (poverty) and treatment. Because geographic differences in survival can result from individuals with similar prognostic factors living in the same areas (e.g. areas with a high rates of late stage CRC or areas with high poverty) it is important to adjust for well known prognostic factors. Such an approach allows one to determine whether the adjustment factors may be contributing causes of the survival disparities. In several studies, geographic disparities in cancer survival persisted despite adjusting survival time for age and stage at diagnosis, two important prognostic factors [21,22].

While previous research has examined a range of determinants of CRC survival, only one study, to our knowledge, has explored geographic variation [22]. This study observed several statistically significant areas in California and Los Angeles County with shorter or longer CRC survival after adjusting patient survival time for age and stratifying by stage. Black patients were more likely to reside in the areas with significantly worse survival, and these lower survival areas were more likely to have high levels of poverty. Additional studies that consider geographic variation in CRC survival are needed to increase our understanding of local community influences that may affect a person's chance of surviving cancer and allow for targeted interventions to population groups at greatest risk of poor outcomes.

In this study we use a recently developed extension of the spatial scan statistic for analyzing time-to-event data to identify whether the survival of CRC patients diagnosed in New Jersey varies by place of residence after adjusting survival times for disease and patient factors. We sought to answer several questions. First, are there areas in New Jersey where CRC survival is significantly longer or shorter than the statewide experience after adjusting survival times for significant prognostic factors (sex, age and stage at diagnosis) and, if yes, what are the approximate locations of these areas? We specifically adjusted patient survival times for age and stage at diagnosis in the initial analysis because we wanted to avoid survival effects resulting from statewide geographic variation in these important prognostic factors, which have been previously documented in New Jersey [25]. Second, if significant geographic differences in CRC survival are detected and we additionally adjust survival times for race/ethnicity and area socioeconomic deprivation, do these geographic survival disparities persist and to what extent are they attenuated?

## Methods

### Data source

CRC cases used for this analysis were obtained from the New Jersey State Cancer Registry (NJSCR), a population-based registry that has collected cancer incidence data since 1979. The NJSCR serves the entire state of New Jersey, which is estimated to have a population of 8.6 million people. The NJSCR has reporting agreements with six other states so that New Jersey residents diagnosed outside the state can be identified. The NJSCR is a participant in the National Cancer Institute's (NCI) Surveillance Epidemiology and End Results (SEER) Program which requires high standards of data quality, as judged by timeliness, completeness and accuracy.

### Study population

Our initial study population consisted of all New Jersey residents reported to the NJSCR with a histologically confirmed, first primary, invasive tumor of the colon or rectum (ICD-O C18.0–C20.9, C26.0, excluding histologies 9590–9989) [26] diagnosed during the period from January 1, 1996 through December 31, 2003 (N = 35,886).

The NJSCR uses NCI SEER summary stage to categorize the stage at diagnosis for each tumor site. SEER summary stages at diagnosis are localized to the primary tumor site, regional by lymph node involvement, regional by lymph nodes and direct extension, and distant metastases [27]. We excluded CRC cases diagnosed at the distant stage because in New Jersey the 5-year relative survival rate for these cases is less than 10% and varies little by race/ethnicity and census tract poverty [28,29]. We also excluded cases with missing stage because a main purpose of this analysis was to study the effects of adjusting for stage on colorectal survival. Therefore only cases diagnosed at a local or regional stage were included. For these patients, there is a substantial chance of cure with appropriate treatment and patient follow-up; the five-year relative survival rate is about 79%. The numbers of patients in each stage group are shown in Table 1.

**Table 1:** Characteristics of first primary invasive colorectal cancer cases in New Jersey, 1996–2003

	Registry Population (N = 35,886)		Survival Analysis (N = 25,040)	
	n	%	n	%
*Age (mean) ± SD*	69.8 ± 13.2		69.1 ± 13	
				
*Gender*				
Male	17,611	49.1	12,423	49.6
Female	18,275	50.9	12,617	50.4
				
*Race/Ethnicity*				
Non-Hispanic White (NHW)	29,194	81.4	20,620	82.4
Non-Hispanic Black (NHB)	3,732	10.4	2,464	9.8
Hispanic	2,040	5.7	1,444	5.8
API	720	2.0	512	2.0
Other, Not Specified	200	0.56	0	0.0
				
*SEER Summary Stage*				
Localized	11,816	32.9	11,308	45.2
Regional, direct extension	6,345	17.7	6,001	24.0
Regional, lymp nodes only	2,882	8.0	2,780	11.1
Regional direct extension and regional lymph nodes	4,848	13.5	4,658	18.6
Regional, Nos	313	0.87	293	1.2
Distant	6,212	17.31	0	0
Missing	3,470	9.7	0	0
				
*Death Certificate Only Cases*	397	1.1	0	0
				
*Geocoding level/Census Tract Assignment*				
Full street address	33,115	92.3	23,881	95.4
Zip code imputed to census tract	1,569	4.4	888	3.6
Zip codes with only one census tract	364	1.0	271	1.1
Not Geocoded/invalid addresss or zip code	838	2.3	0	0
*Census Tract Poverty*				
% Living below poverty				
(Low)Q1 (0–3.0)	9087	25.3	6624	26.5
Q2 (3.1–5.5)	10419	29.0	7475	29.9
Q3 (5.6–12)	9217	25.7	6579	26.3
(High)Q4 (>12)	6325	17.6	4362	17.4
Missing Census Tract Poverty	838	2.3	0	0

Source: Data from the New Jersey State Cancer Registry, 2007; U.S. Census Bureau, 2000

### Analytical Variables

The following individual-level variables known or suspected to be associated with CRC survival were included in the analysis: age at diagnosis, race/ethnicity group (non-Hispanic white, non-Hispanic black, Hispanic, Asian/Pacific Islander), sex (male, female), and stage at diagnosis (local, regional direct extension, regional lymph nodes only, regional extension and nodes, regional NOS). Cases with unknown or other race/ethnicity and those with an unknown address were excluded. A total of 25,040 cases were used in the analyses (Table 1).

Cases were geocoded to the residential address at time of diagnosis. Of the geocoded cases, 23,762 (94.8%) were successfully assigned to census-tracts using the street segment of the address. We assigned the remaining 1,278 cases (5.2%) to imputed census-tracts based on the postal delivery area to avoid potential geographic selection bias resulting from excluding cases not successfully geocoded [30]. Census tracts were chosen as the units of analysis because they are small, relatively permanent statistical subdivisions of a county and are designed to be homogenous with respect to population characteristics, economic status, and living conditions. New Jersey has 1,951 census tracts which, on average, contain 4,300 persons.

We selected the 2000 U.S. Census tract poverty rate (the percentage of population below the poverty line) as the area-based socioeconomic measure of deprivation [31]. The literature suggests that area-based socioeconomic characteristics play an important role in affecting a person's health, independent of that person's individual socioeconomic status [9,32-34]. Area-based measures such as the poverty rate have been shown to capture the discrepancies in distribution of neighborhood social and economic conditions that affect residents[34,35]. Despite the presence of many single and composite deprivation measures, we specifically chose poverty because several studies, including those completed using New Jersey cancer registry data, [25,29] have found census-tract poverty to be a useful measure of economic deprivation of area-based socioeconomic variations in cancer incidence, survival and other health outcomes [34,36,37]. The census tract poverty measure was grouped into quartiles (Q1 (0–3.0), Q2 (>3.0–5.5), Q3 (>5.5–12), Q4 (>12.0)) based on the statewide census-tract distribution of this measure.

### Patient Follow-up

The NJSCR conducts passive and active follow-up of cancer patients for vital status using linkages with state and national death files, state taxation files, hospital discharge files, Medicare and Medicaid files, Social Security Administration Services for Epidemiologic Researchers, motor vehicle registration records, and by contacting hospitals and physicians' offices. Patients were followed until their death or until December 31, 2006, which is also the date of censoring for patients who were last known to be alive. Completeness of vital status follow-up for CRC cases through December 31, 2006 is around 96%. We excluded 496 cases with no follow-up time. Over 80% percent of these cases included cases reported from death certificates where the date of diagnosis and date of death were the same. Underlying cause of death was abstracted from death certificates, and identified as due to colon or rectal cancer according to the International Classification of Diseases (1996–1998 (ICD-9) 153.0–154.1, 159 and 1999–2003 (ICD-10) C18–C20, C26).

### Statistical analyses

We used cancer (cause) specific survival as our primary measure of patient survival. Cancer-specific survival (or equivalently cause-specific mortality) is a net survival measure representing survival of a specified underlying cause in the absence of other causes of death [38]. This measure is based on the assumption that deaths from a specified cause are independent of deaths from other causes. It has been shown to be a useful measure for cancer control when comparing cancer survival between racial/ethnic or socioeconomic groups or between geographic areas where death due to other causes may differ; the NCI refers to this measure as a "policy based statistic" [9,22,38-42]. Cancer-specific survival is also consistent with population-based cancer mortality rates, which are also based on the underlying cause of death.

We specified CRC as the underlying cause of death for this analysis. Patient survival times were measured in months from the date of diagnosis and were censored at the date of death from causes other than CRC, the date a patient is lost to follow-up or at the end of the follow-up period, December 31, 2006 (whichever occurred first). The Kaplan-Meier estimator was used to estimate CRC-specific survival rates for race/ethnicity, sex, and poverty quartiles by stage at diagnosis, and each were compared with the log-rank test. Five-year CRC-specific survival rates and associated 95% confidence intervals (CI) were also computed based on Kaplan-Meier survival curves.

### Geographical Analyses

We applied the exponential model based spatial scan statistic using SaTScan software (v.7.02) to determine whether there is geographic variation in CRC survival without any a priori assumptions regarding the location or size of possible variation [43,44]. Survival time was modeled using an exponential probability distribution, comparing the mean survival times of patients in a geographical area (*θ*in) with that of patients outside that area (*θ*out). The entire study region is examined for significant deviation in survival by using a circular scanning window that varies in size from 2 cases to a maximum of 50% of the cases. We choose a circular scanning window because this shape has been shown to be effective at highlighting general areas or regions of concern. For each circular window the maximum likelihood method was used to test for deviations from the null hypothesis that the mean CRC survival time of cases inside and outside the scanning window are equal (Ho: *θ*in =*θ*out; Ha:*θ*in ≠ *θ*out). Finally, a Monte Carlo permutation was used to evaluate statistical significance and adjust for multiple testing by permuting survival time and censoring indicators among locations. A more thorough review of the exponential based spatial scan statistic and the permutation test can be found elsewhere [45].

Before applying the spatial scan statistic to search for areas with short or long survival, CRC patient survival times were adjusted for covariates using three separate fixed effects exponential regression models. The three models included the following covariates: (1) sex, age, and stage at diagnosis; (2) sex, age, stage at diagnosis, and race/ethnicity; (3) sex, age, stage at diagnosis, race/ethnicity, and census-tract poverty quintiles. The adjustment produces expected survival times based on the specified explanatory factors. Details of such adjustments have been described elsewhere [22,45].

Spatial scan statistic analysis was conducted separately for each of the adjusted survival time datasets, as described above. We identified all statistically significant (p =< 0.05) areas (circles) with shorter or longer than expected survival regardless of scanning window location or size and mapped the results using a nested circle approach proposed by Boscoe et al. (2003), which is summarized as follows. First, the survival areas detected were stratified into equal intervals of risk (observed/expected CRC deaths). Within each risk interval, the area with the highest likelihood ratio (lowest p value) was mapped. Areas with lower likelihood ratios were also mapped if they did not overlap any previously mapped area within the same risk interval. Mapping was completed using ArcGIS 9.3 software [46].

For areas with statistically significant shorter or longer survival, we reported the total cases, observed deaths, ratio of observed/expected CRC deaths (obs/exp) as defined by SaTscan, the percent of cases by race/ethnicity, and the average percent of census-tract poverty among the cases. The expected CRC deaths were based on a comparison of individual time (either survival time or censoring time) with the mean survival time – if the observed individual time was smaller than the mean survival time, it was considered a death. We also reported adjusted 5-year survival rates and 95% confidence intervals (CI) inside and outside each of the detected survival areas using a method by Zhang et al. (2007) which uses Cox regression estimates to adjust survival for selected covariates. Adjusted survival time and 5-year survival rates were calculated using SAS, version 9.1[47]

## Results

### Descriptive Analyses

Table 1 describes the overall population of CRC cases reported to the NJSCR from 1996 through 2003, as well as the population subset used for the study. Among the cases used in the study 82.4% were non-Hispanic white, 9.8% were non-Hispanic black, 5.8% were Hispanic and 2.0% were Asian Pacific Islander (API). Cases ranged in age from 18 to 101 with an average age of 69. Approximately 45% of the cases were diagnosed at the local stage and 55% at the regional stage.

Among the 25,040 cases included in the study, 4,858 died from CRC and 20,182 cases were right censored (1,327 lost to follow-up; 6,070 non-CRC deaths; 12,785 alive at the end of follow-up [December 31, 2006]). Characteristics of the local and regional stage CRC cancer cases used for the analyses are presented in Table 2. The five-year CRC-specific survival rate was 90.7% for local stage cases, 70.4% for regional stage cases, 79.6% overall. Race/ethnicity effects on survival were statistically significant (log-rank P < 0.001). The five-year survival rate was 83.2% in non-Hispanic whites, the highest among all racial/ethnic groups, compared with the lowest 75.6% in blacks. Area poverty gradients were observed in survival rates for both local and regional cases and the effects were statistically significant (log-rank P < 0.001).

**Table 2:** Colorectal cancer case characteristics and 5-year survival of localized and regional cases used in the analyses by sex, race/ethnicity, census tract poverty and mean age at diagnosis (1996–2003)

	**Study Population 5-Year Survival (95%CI)**	**Localized Cases 5-Year Survival (95%CI)**	% Cases	**Regional Cases 5-Year Survival (95%CI)**	% Cases
	*n = 25,040*	*n = 11,308*		*n = 13,732*	
*Total population*	79.6 (79.1, 80.2)	90.7 (90.1, 91.2)	45.2	70.4 (69.5, 71.2)	54.8
*Sex*					
Male	80.2 (79.5, 81.0)	90.9 (90.0, 91.7)	45.9	71.1 (69.8, 72.7)	54.1
Female	79.0 (78.2, 79.4)	90.4 (89.6, 91.2)	44.4	69.8 (68.6, 71.0)	55.6
P value from Log-rank	0.01	0.79		0.03	
*Race/Ethnicity*					
Non-Hispanic White	83.2 (79.6, 86.9)	92.2 (87.9, 96.5)	45.5	77.6 (72.4, 82.8)	54.5
Non-Hispanic Black	75.6 (73.9, 77.6)	87.6 (85.4, 89.7)	45.1	65.7 (62.9, 68.6)	55.0
Hispanic	79.1 (76.8, 81.4)	90.1 (87.6, 92.6)	43.7	70.5 (67.1, 74.0)	56.2
API	80.0 (79.4, 80.6)	91.0 (90.3, 91.6)	37.1	70.7 (69.8, 71.7)	62.9
P value from Log-rank	<0.0001	0.009		0.0002	
					
*Census Tract Poverty*, % Living below poverty					
(Low)Q1 (0–3.0)	81.8 (80.8, 82.8)	92.2 (91.2, 93.2)	45.2	73.2 (71.7, 74.8)	54.8
Q2 (3.1–5.5)	80.9 (80.0, 81.9)	91.8 (90.8, 92.8)	45.6	71.6 (70.1, 73.1)	54.4
Q3 (5.6–12)	78.9 (77.8, 80.0)	89.4 (88.2, 90.6)	45.4	70.0 (68.4, 71.6)	54.6
(High)Q4 (>12)	74.9 (73.4, 76.3)	87.9 (86.3, 89.5)	44.0	64.4 (62.3, 66.6)	56.0
P value from Log-rank	<0.0001	<0.0001		<0.0001	

Source: Data from the New Jersey State Cancer Registry, 2007; U.S. Census Bureau, 2000; CI = Confidence Interval API = Asian Pacific Islander

5-year colorectal cancer specific survival rates based on Kaplan-Meier estimates

### Geographic Analyses

**Figure 1:**
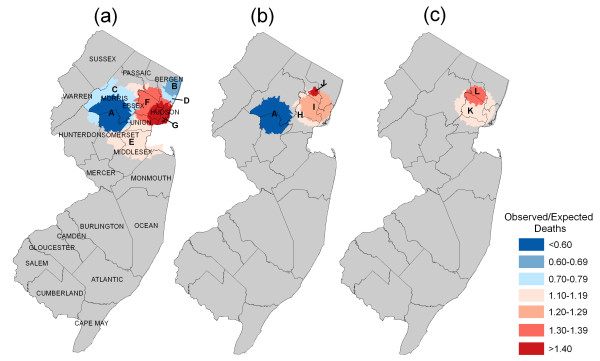
**Areas with statistically significant shorter or longer than expected survival following diagnosis of colorectal cancer adjusted for: (1) sex, age, stage at diagnosis (2) sex, age, stage at diagnosis, race/ethnicity (3) sex, age, stage at diagnosis, race/ethnicity, census tract poverty.** Area characteristics are summarized in Table 3.

In the geographic analyses, several regions of New Jersey showed statistically significant differences in CRC survival. Table 3 describes the survival characteristics of the areas having significantly shorter or longer survival from each of the models. Figure 1 illustrates the survival locations and related ratios of observed to expected CRC deaths.

**Table 3:** Case characteristics and adjusted 5-year survival rates for areas with shorter or longer than expected survival as defined by the spatial scan statistic.

Survival time adjusted for:	Study Area^a^	Total Cases	Obs deaths	O/E deaths	P-value	AVG % CT Poverty^b^	% NHW	% NHB	% H	% A	Adjusted 5-year survival^b ^(95% CI) inside survival area^d^	Adjusted 5-year survival^c ^(95% CI) outside survival area^d^
***a. ****Age, sex, stage*												
	A	1,252	188	0.72	**0.040**	3.4	91.1	2.2	3.8	2.9	84.2 (82.1, 86.3)	78.9 (78.4, 79.5)
	B	486	61	0.54	**0.010**	3.2	91.6	3.3	1.9	3.3	88.3 (85.4, 91.3)	79.1 (78.5, 79.5)
	C	1,713	271	0.75	**0.047**	5.9	82.6	5.3	6.8	5.4	83.6 (81.8, 85.5)	78.9 (78.3, 79.5)
	D	954	133	0.67	**0.043**	4.4	80.7	8.2	6.2	4.9	85.6 (83.2, 88.0)	79.0 (78.4, 79.5)
	E	10,113	2,124	1.10	**0.008**	9.3	73.6	14.4	9.8	2.3	77.5 (76.7, 78.3)	80.4 (79.7, 81.1)
	F	5,804	1,324	1.24	**<0.001**	12.2	65.5	19.5	12.9	2.1	75.2 (74.0, 76.3)	80.4 (79.8, 81.0)
	G	2,771	666	1.40	**<0.001**	17.8	50.1	27.7	19.8	2.4	73.1 (71.5, 74.9)	80.0 (79.4, 80.5)
												
***b. ****Age, sex, stage, race/ethnicity*												
	A	1,252	188	0.74	0.424						83.7 (81.5, 85.9)	78.9 (78.4, 79.5)
	B	486	61	0.56	**0.048**						87.9 (84.9, 90.9)	79.0 (78.5, 79.5)
	C	1,713	271	0.76	0.164						83.3 (81.5, 85.2)	78.9 (78.3, 79.4)
	D	954	133	0.68	0.093						85.4(83.1, 87.8)	78.9 (78.4, 79.5)
	E	10,113	2124	1.07	0.809						77.9 (76.9, 78.6)	80.1 (79.5, 80.8)
	F	5,804	1324	1.17	**<0.001**						76.0(74.5, 76.9)	80.2 (79.6, 80.8)
	G	2,771	666	1.25	**0.002**						74.5 (72.4, 76.0)	79.8 (79.2, 80.3)
	H	5,772	1,315	1.18	**<0.001**	12.2	65.5	19.5	12.9	2.1	75.7 (74.6, 76.9)	80.4 (79.8, 81.0)
	I	4,621	1,072	1.20	**<0.001**	12.9	63.6	21.3	12.9	2.2	75.4 (74.1, 76.7)	80.2 (79.7, 80.8)
	J	386	125	1.63	**0.037**	16.3	57.5	23.3	18.1	1.0	71.4 (67.1, 75.7)	79.3 (78.9, 80.0)
												
***c. ****Age, sex, stage, race/ethnicity, CT poverty*												
	A	1,252	188	0.78	0.987						83.0 (80.8, 85.3)	79.0 (78.4, 79.5)
	B	486	61.0	0.60	0.398						87.3 (84.2, 90.5)	79.0 (78.5, 79.5)
	C	1,713	271	0.78	0.495						83.1 (81.3, 85.0)	78.9 (78.3, 79.4)
	D	954	133	0.72	0.701						84.8 (82.3, 87.3)	78.9 (78.4, 79.5)
	E	10,113	2124	1.06	0.987						77.9 (77.1, 78.8)	80.0 (79.3, 80.7)
	F	5,804	1324	1.12	0.132						76.3 (75.2, 77.5)	80.0 (79.5, 80.6)
	G	2,771	666	1.13	0.987						75.7 (73.9, 77.5)	79.6 (79.1, 80.2)
	H	5,772	1315	1.13	0.107						76.3 (75.0, 77.4)	80.0 (79.4, 80.6)
	I	4,621	1072	1.14	0.146						76.2 (74.8, 77.4)	79.9 (79.3, 80.5)
	J	386	125	1.54	0.344						72.6 (68.3, 76.8)	79.3 (78.7, 79.8)
	K	5,404	1,224	1.15	**0.017**	11.3	67.2	20.7	9.9	2.2	76.2 (75.0, 77.4)	80.3 (79.7, 81.0)
	L	1,316	327	1.32	**0.048**	8.1	78.9	11.1	9.0	1.1	74.6 (72.2, 76.9)	79.5 (79.0, 80.0)

Geographic locations illustrated in figure 1.

^a ^Short or long survival areas detected by the spatial scan statistic are illustrated in Figure 1.

^b ^Averages for each area are based on the census tract values assigned to the cases.

^c ^Directly adjusted 5 year colorectal cancer specific survival rates are based on a Cox regression model adjusted for specified covariates. The method adjusts survival time by covariates to adjust for any imbalance of CRC case characteristics inside and outside defined survival area (e.g All CRC cases inside area A versus all CRC cases outside area A).

^d ^Inside refers to the adjusted survival time of all CRC cases inside the specified survival area (e.g Cases inside detected area A). Outside refers to the adjusted survival time of all CRC cases outside the specified survival area (e.g. Cases outside detected area A).

P-value from spatial scan statistic Monte Carlo permutation test.

Statistically significant departures from the statewide rates occurred only in the northern part of the state. Longer than expected survival in areas A and B (suburbs of Morris and Somerset counties) and in areas C and D (a densely populated portion of Bergen County) correspond to predominantly high-income white neighborhoods (Figure 1a). Residents of these areas had a lower-than-expected risk of CRC death than elsewhere (O/E = 0.72, 0.54, 0.75, 0.67 for A, B, C, D, respectively, with p-values < 0.05); and the adjusted 5-year survival rates ranged in these areas from 88.3% to 83.6%, several percentage points higher than elsewhere (approximately 80%) (Table 3a).

Shorter survival times were estimated among cases in areas E, F and G, nested in the north central part of the state. The worst outcomes were found in area G, in the cities of Newark, Elizabeth, and East Orange (Essex County) and Union and Jersey City (Hudson County), predominantly low-income black and Hispanic neighborhoods (Figure 1a). The risk of dying from CRC among persons living in area G was estimated 1.4 times greater than elsewhere (p < 0.001), and the area had a 73.1% survival rate compared with 80% elsewhere in the state (Figure 1a).

Additional adjustment of survival times for race/ethnicity resulted in several areas becoming non-significant and three new areas, all of which overlap previously defined areas – H and I are attenuated versions of areas F and G, and J is an elevated version of part of area F (Table 3b). Area B remained the only area with significantly longer than expected survival. Of the newly defined areas with shorter than expected survival, area J, located partially in Passaic City, a relatively low income and largely white and Hispanic area, had the worst survival (Figure 1b). The risk of dying from CRC in this area was estimated to be 1.6 times greater than among the other cases in the state (Table 2). The adjusted 5-year survival rate was 71.4% (95% CI 61.1, 75.7) compared with 79.3% (95% CI 78.9, 80.0) elsewhere.

After additional adjustment of survival times for census-tract poverty, several more areas became non-significant, and the risk of dying from CRC was further reduced in areas previously detected with shorter survival (Table 3c). No significantly longer than expected survival areas remained. Two remaining areas with significantly shorter than expected survival (K and L) were located in the same region as the previously defined areas (O/E = 1.2 and 1.3 for K and L, respectively) (Figure 1c).

## Discussion

Our findings suggest that survival of CRC patients diagnosed in New Jersey varies by place of residence after adjusting for disease and patient characteristics. Geographic analysis based on age and stage adjusted survival times detected several areas in the northern part of the state where CRC survival outcomes were significantly different than expected. Survival disparities persisted in some areas even after adjusting patient survival times for race/ethnicity and area socioeconomic deprivation, as defined by census-tract poverty.

Regional demographics and patient characteristics provide some evidence that the initial results based on age-stage adjusted survival might reflect geographical concentrations of patients who can be presumed at greater or less risk of poor outcomes regardless of age and stage at diagnosis – blacks and persons living in poor areas may be at greater risk of poor outcomes compared with whites and persons living in wealthy areas (Table 3)[29,40,48,49]. Areas detected with the best survival, for example, were found in predominately high income white neighborhoods in Morris, Somerset and Bergen counties; whereas areas with the worst survival were found in mostly low income, racially diverse neighborhoods in several large cities in Essex and Union counties. There are numerous characteristics of poor neighborhoods that could impede patient survival such as high unemployment, poor education, health impairing environmental exposures, substandard housing and limited access to resources and information.

Our age-stage adjusted survival estimates were consistent with findings of Huang et al. (2007) who completed a similar study using CRC data from California. They found several statistically significant areas in Los Angeles (LA) County with shorter or longer CRC survival after adjusting patient survival time by age and stratifying by stage. The shorter survival areas in LA County, like those detected for New Jersey, had both a higher percent of black cases and a higher percent of cases living in impoverished areas than the longer survival areas. In another study that used similar methods, but analyzed prostate cancer survival, Gregorio et al. (2007) also noted significant geographic variation after applying age-stage adjusted survival times.

Adjustment for patient's race/ethnicity and area socioeconomic deprivation further reduced survival disparities for several areas, but two areas remained significant with worse-than-expected outcomes. These results suggest race/ethnicity and area socioeconomic deprivation does affect outcomes in some locations in New Jersey, while one area in particular remains unexplained. Further research is needed to identify the causal factors that mediate this relationship.

It is unclear why areas of worse than expected CRC survival remain unexplained after adjusting for area socioeconomic deprivation. Possibilities include a local problem of access to health care or a pattern of care at one or more hospitals. Such patterns have been documented in the Dartmouth Atlas of Health Care project [50]. For stage III colon cancer, Etzioni et al. (2008) found that the likelihood of receipt of chemotherapy was influenced by referral patterns, hospital volume, and the presence of a cancer program approved by the American College of Surgeons' Commission on Cancer [51]. There may be other individual factors that contribute to these survival differences. For example, it could be related to modifiable risk factors (e.g smoking, diet, exercise) or comorbidity that are often themselves geographically structured [24]. Also since these areas are ethnically diverse, with substantial immigrant populations; it is possible that language barriers may affect access or coordination of care [52]. It is also possible that the adjustments for race/ethnicity and socioeconomic deprivation are inadequate or incomplete.

We have identified significantly divergent areas of CRC survival in New Jersey after adjusting for important prognostic factors, including age and stage at diagnosis. The next focus of investigation could be comparing their differences in comorbidity status as well as medical care in terms of access, utilization and quality. Such analysis could be completed for persons 65 years and older using the SEER-Medicare linked database which includes registry data and Medicare claims for covered health care services, including hospitalizations. Schootman et al. (2009) used this database to examine geographic patterns of breast cancer survival in several geographic areas[23]. It would also be important to examine other determinants of CRC risk and survivorship such as diet and exercise. Interviewing cancer survivors in these locations about their experiences combined with medical chart reviews may lead to clarification of groups at greatest risk of dying from CRC and provide explanations to geographical patterns of CRC survival. Perhaps future analysis might reveal that areas with longer than expected survival may highlight protective effects such as social support and/or clinical advancements that warrant replication in other places.

Applying the spatial scan statistic as we did in this study to adjusted survival times allows a better understanding of the extent to which geographic patterns of CRC survival can be explained by important risk factors. Documenting risk of death (observed vs expected) by geographic area after each covariate adjustment and tracking changes in risk provides a useful approach, analogous to the methods used in non-spatial statistics (e.g., Cox regression). While traditional non-spatial analysis provides greater clarity as to the precise contribution of each risk factor, this complementary approach has the advantage of highlighting specific geographic locations.

Using the 'nested circles' approach to map areas detected from the spatial scan statistic provides a greater degree of information about the risk of death from CRC among significant localized excess contained within a broader region of deficit. Typically, studies using the spatial scan statistic document only the areas that are significant, have the highest likelihood ratios, and do not overlap. As Boscoe et al. explain, this approach tends to identify large geographic areas with large populations, but small elevations in risk because these areas have the highest statistical power[53]. Changing the maximum scanning window size in the software can control for this, but the optimal size is not obvious. Also, selecting the final maximum window size on previous analyses can lead to pre-selection bias. In our study, areas E and D would have been the only areas detected if we followed typical practice and set the scanning window to a maximum of 50% of the cases and did not allow for geographic overlap. This has important public health implications if the areas detected will be used for targeted intervention.

For cancer control and prevention activities it is also important to acknowledge two further caveats related to the interpretation of our results. First, the significantly better or worse survival areas detected in our study may not be circular and are based on an identification procedure that relies on circular scanning windows. While circles are effective at highlighting general areas or regions of concern the boundaries of these areas are always approximate. Other shapes could have been employed (e.g. elliptical) and this would have likely resulted in somewhat different boundaries. Second, it is also important to consider the variation within the detected clusters and to remember that risk within these areas may not be evenly distributed. Future work using the spatial scan statistic should consider displaying maps of smoothed survival rates (descriptive or model based) beneath the statistically significant clusters [54]. Doing so would provide additional information that could be helpful to assist with the generation of hypotheses about underlying causes of survival disparities. Approaches for mapping geographic variation of patient survival have been demonstrated by Banerjee et al. (2003) and Lawson [55,56].

The results of this study need to be evaluated in light of a number of important limitations. First, by using CRC-specific survival we are presuming accuracy of the underlying cause of death on death certificates [57]. The extent to which misclassification of underlying cause of death occurred and the impact it had on our findings cannot be determined. It has been reported, however, that when deaths from colon cancer or rectum cancer are combined the accuracy of coding CRC as an underlying cause of death on death certificates is around 93 percent [58]. However, little is known about whether the accuracy of cause of death on death certificates varies by geography.

Another limitation is related to our inability to assess competing risks. Competing risks occur when there are at least two possible ways a patient can die (patients in our study could die from CRC or some other cause). When using cause-specific survival the assumption is deaths from a specified cause (e.g. CRC) are independent of deaths from other causes – thus we are assuming the absence of competing risks. If the independence assumption is not met, a bias could result because cases who are censored are more likely to die than non-censored cases [59,60] The main reason we could not assess competing risks was because we did not have comorbidity information or individual risk factors (e.g smoking). Instead we conducted sensitivity analyses of our geographic results using the following censoring scenarios: [60] (1) all patients previously censored because of deaths from other causes are assumed to die of CRC (all cause-survival); (2) all patients previously censored because of deaths from other causes survive to the end of follow-up (December 31, 2006); (3) a randomly selected subset of 5% (or 10%, 20%, 30%, 40%, 50%) of patients previously censored because of deaths from other causes are assumed to die of CRC. These scenarios allow us to consider what Kleinbaum and Klein refer to as "worst-case violations of the independence assumption" [60]. For each scenario, we detected survival disparities in approximately the same locations as the CRC-specific analysis; however scenario (1) detected a significant area of shorter than expected survival in the southwestern part of the state. Similarities between the different censoring scenarios and our results suggest minimal bias related to the independence assumption.

A third potential limitation is the geographic distribution of cases censored because they were lost to follow-up (e.g. migration). While there were more cases lost to follow-up inside the shorter and longer survival areas compared to outside each of these areas, the differences were minimal (the maximum difference was around 2 percent) and would likely have nominal impact on our findings. Also a review of the proportion of cases censored because of non-CRC deaths inside and outside the shorter survival areas deaths indicated no significant differences.

Further limitations include a lack of important patient information available from the NJSCR. For example, incomplete CRC treatment data at the NJSCR limited our ability to use this information to determine its impact on survival differences. Furthermore, the NJSCR only collects information on first course of treatment. Patient insurance data was not included because it was only required by the NJSCR starting in 1999. And the NJSCR and the SEER programs do not collect information about co-morbidity or lifestyle risk factors that may be associated with cancer incidence and prognosis (e.g. obesity, smoking, diet, and alcohol).

One additional caveat concerns how missing data excluded from our study may bias our findings [61,62]. Cancer registry data is often not missing at random, but varies by age, race/ethnicity, socioeconomic status and geography[63,64]. Among cases excluded due to incomplete geocoding (2%) there were no statistically significant differences by race/ethnicity, but these cases were more likely to be older than 75 years of age and missing stage information. Because there were so few cases with incomplete geocoding it is unlikely to have influenced our results. The greatest number of cases excluded were due to missing stage of disease at diagnosis (approximately 9.6%). Cases typically lack stage information due to medical decisions, lack of information in the medical record due to a superficial workup, or because they were obtained from death certificates only (DCO). Stage which is a proxy for prognosis is often missing in population based registries which, not only make the geographic picture incomplete, but may introduce bias [63]. Treatment information as well as information on stage is related to socioeconomic status [65], and missing stage has been shown to be higher among blacks than whites in central cancer registry data [64]. In our study we found statistically significant differences among cases without stage information by race/ethnicity (Whites, 9.3% versus blacks, 10.8%), age at diagnosis (<75, 8.6% versus >75, 17.8%) and area poverty (Low poverty, 9% versus high poverty 10.8%). Survival estimates for cases missing stage were more similar to regional stage disease than local or distant stage disease. Because the profile of cases missing stage were more similar to those at greatest risk of dying from CRC, our survival estimates could be slightly conservative if a substantial number of the cases were local or regional stage and were geographically distributed non-randomly.

Despite several potential limitations, this study is strengthened by the use of histologically confirmed CRC cases followed for up to 10 years from a large population-based SEER cancer registry with a large population (8.7 million people) and socioeconomic and racial diversity. Further strengths are high quality geocoding and the completeness of patient vital status follow-up (95.5%) since the NJSCR uses both active and passive methods.

## Conclusion

In summary, we observed significant differences in age and stage at diagnosis adjusted survival by geographic location among the over 25,000 residents of New Jersey diagnosed with localized or regional stage CRC from 1996 through 2003. Further adjustment for race/ethnicity and area poverty reduced geographic survival disparities but did not completely explain them. These findings suggest that, in areas where adjustment changed the geographic survival patterns and reduced the risk of death, these factors may be contributing causes of the disparities. Conversely, geographic disparities that persist after adjustment likely indicate areas of unexplained, and potentially amendable, variation. Further studies need to focus on identifying specific pathways by which local factors and area socioeconomic deprivation explain geographic survival disparities.

Our use of the recently developed exponential based spatial scan statistic to examine geographic variation in CRC survival demonstrates how researchers and public health practitioners can apply this method to monitor cancer survival disparities, evaluate the effectiveness of statewide or locally based interventions and generate hypotheses about the underlying causes of geographic disparities in cancer survival. To our knowledge the exponential based statistic has only been applied to cancer survival data, but its usefulness for analyzing time-to-event makes it suitable for other applications including disease remission, cure or cessation of behavior, or hospital discharge time.

## Competing interests

The authors declare that they have no competing interests.

## Authors' contributions

KAH conceived the study and wrote the manuscript. Both KAH and XN performed the analyses. FPB provided feedback on the study design, helped interpret the results and reviewed drafts of the manuscript. All authors read and approved the final version of the manuscript.
